# REDfly: the transcriptional regulatory element database for *Drosophila*

**DOI:** 10.1093/nar/gky957

**Published:** 2018-10-17

**Authors:** John Rivera, Soile V E Keränen, Steven M Gallo, Marc S Halfon

**Affiliations:** 1 Center for Computational Research, State University of New York at Buffalo, Buffalo, NY 14203, USA; 2 New York State Center of Excellence in Bioinformatics and Life Sciences, State University of New York at Buffalo, Buffalo, NY 14203, USA; 3 Department of Biochemistry, State University of New York at Buffalo, Buffalo, NY 14203, USA; 4 Department of Biomedical Informatics, State University of New York at Buffalo, Buffalo, NY 14203, USA; 5 Department of Biological Sciences, State University of New York at Buffalo, Buffalo, NY 14203, USA; 6 Department of Molecular and Cellular Biology and Program in Cancer Genetics, Roswell Park Cancer Institute, Buffalo, NY 14263, USA

## Abstract

The REDfly database provides a comprehensive curation of experimentally-validated *Drosophila* transcriptional *cis*-regulatory elements and includes information on DNA sequence, experimental evidence, patterns of regulated gene expression, and more. Now in its thirteenth year, REDfly has grown to over 23 000 records of tested reporter gene constructs and 2200 tested transcription factor binding sites. Recent developments include the start of curation of predicted *cis*-regulatory modules in addition to experimentally-verified ones, improved search and filtering, and increased interaction with the authors of curated papers. An expanded data model that will capture information on temporal aspects of gene regulation, regulation in response to environmental and other non-developmental cues, sexually dimorphic gene regulation, and non-endogenous (ectopic) aspects of reporter gene expression is under development and expected to be in place within the coming year. REDfly is freely accessible at http://redfly.ccr.buffalo.edu, and news about database updates and new features can be followed on Twitter at @REDfly_database.

## INTRODUCTION

Transcriptional regulation is a fundamental biological process, but the identification, characterization, and incorporation into the genome annotation of metazoan transcriptional *cis*-regulatory sequences is still surprisingly limited relative to knowledge of other genomic features. A marked exception is the *Drosophila melanogaster* genome, where a dedicated effort has been made to curate the results of over three decades of molecular and genetic characterization of transcriptional *cis*-regulatory modules (CRMs) such as enhancers, silencers, and proximal promoter sequences in the form of the *R*egulatory *E*lement *D*atabase for *Drosophila*, or REDfly (in keeping with previous usage (1,2) we use CRM as a generic term to refer to transcriptional regulatory elements located outside of the core promoter region which regulate gene expression in a spatio-temporal manner). REDfly was established in 2006 (3) and merged with the FlyReg DNaseI footprint database (4) in 2008 to create a comprehensive database of *Drosophila* regulatory sequences, both CRMs and transcription factor binding sites (TFBSs) (2). REDfly's goal is to provide a single source for information on known *Drosophila* CRMs and TFBSs along with their DNA sequences, their associated genes, and the expression patterns they direct.

Since its inception, REDfly has grown from a collection of ∼600 CRMs to one of close to 17 000, with records of more than 2000 TFBSs (Table 1). As the most comprehensive available resource for curated metazoan regulatory sequences, REDfly has proven valuable for many research purposes, including the study of CRM biology (e.g. 5,6–13); interpretation of genomic data from ChIP and other chromatin-based studies (e.g. 14,15–22); empirical and computational CRM discovery (e.g. 23,24–32), including cross-species CRM discovery in and annotation of non-drosophilid insect genomes (33,34); TFBS prediction (35); regulatory network modeling (e.g. 36,37–41); and CRM evolution (e.g. 42,43–48), plus numerous single-locus studies of the regulation of specific genes.

**Table 1.** Comparison of REDfly contents, August 2011 and September 2018

	REDfly v3.0 (1 August 2011)	Current (1 September 2018)	Fold change
**RCs**			
Total	1351	23 193	17.2×
From in vivo reporter genes	1319	12 460	9.4×
From cell culture assays	18	10 445	580.3×
Associated genes	344	831	2.4×
**CRMs**			
Total	1046	16 768	16.0×
From in vivo reporter genes	1027	6473	6.3×
From cell culture assays	11	10 273	933.9×
Associated genes	327	756	2.3×
**TFBSs**			
Total	1662	2209	1.3×
Transcription factors	113	179	1.6×
Target genes	142	246	1.7×
**Predicted CRMs**			
Total	0	8175	∞
**Publications curated**			
Total	483	810	1.7×

In this update, we review changes and additions to REDfly since our last published description (1), and highlight exciting forthcoming improvements expected over the next year.

## REDfly DATA CLASSES

### Reporter constructs and inferred CRMs

**Figure 1.**
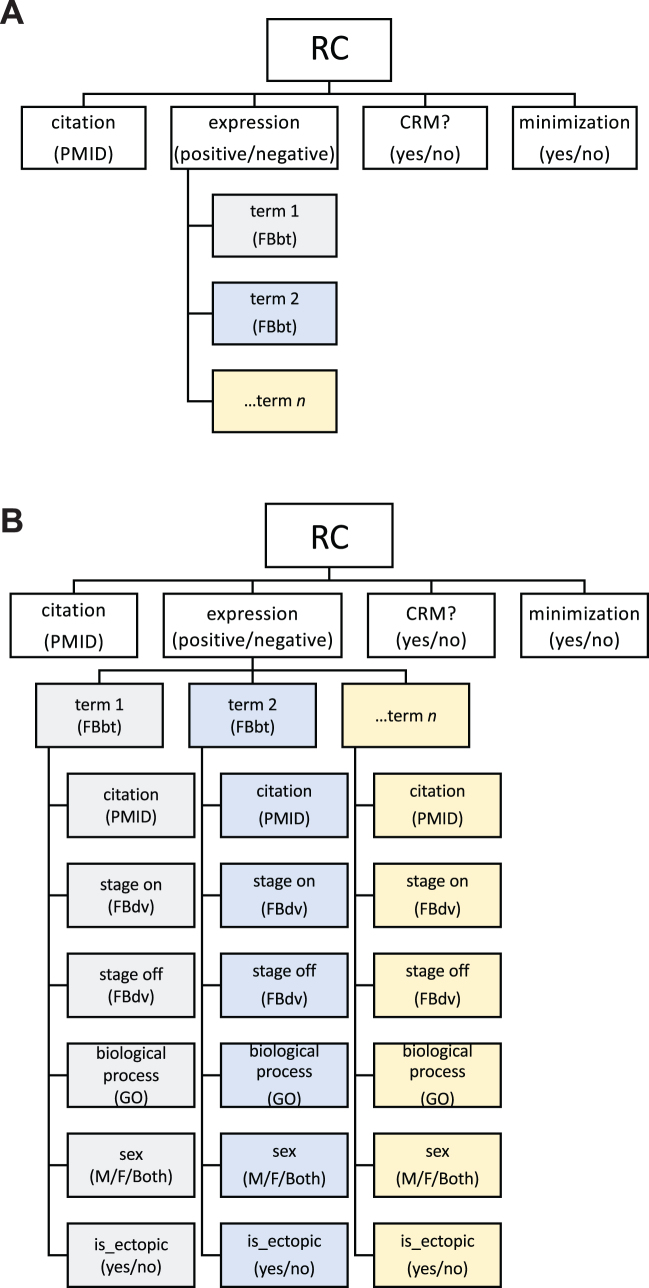
Current and future REDfly reporter construct data models. (**A**) A partial illustration of the current reporter construct (RC) data model, showing an RC with its three basic attributes of *expression, CRM, and minimization*, along with citation information. Citations are in the form of a PubMed_ID number (PMID). For RCs with positive expression, one or more expression terms from the *Drosophila* anatomy ontology (‘FBbt’ terms) are associated with the record. Not included in the schematic are additional associated data such as gene names, relevant figure panels, evidence terms, and others. (**B**) The expanded RC data model under development. In this new model, additional data are associated with each annotated expression pattern, including a citation; the stages at which expression is observed (based on the *Drosophila* development ontology, ‘FBdv’); a biological process (where relevant, e.g. ‘hypoxia’) drawn from the Gene Ontology (‘GO’); the sex in which the expression is observed; and whether or not the expression is ectopic relative to the known pattern of the associated gene (when known). As in panel A, additional associated data such as gene names are not shown in the schematic.

From the beginning, REDfly's philosophy has been to focus on empirically-tested DNA sequences, typically from reporter gene assays in transgenic animals (although other assays are also accepted, e.g. cell-culture-based reporter gene assays). We define all sequences tested in this manner as Reporter Constructs (RCs), each of which has three associated attributes: *expression*; *CRM*; and *minimization* (Figure 1A; see (1) for more details). *Expression* has value ‘positive’ or ‘negative’ and describes whether or not the sequence was observed to drive gene expression in the reporter gene assay. RCs with positive expression have their expression patterns annotated using the *Drosophila* anatomy ontology (49). An RC is considered to be a *CRM* if it is the shortest of a set of nested sequences with identical activity, or the only annotated sequence covering a given set of genomic coordinates. In cases where there are multiple nested RCs, the set of RCs is said to have undergone ‘*minimization*.’

In 2011, we introduced a new class of data, the *inferred CRM* (iCRM). Often, two overlapping sequences have the same regulatory activity *in vivo*, which suggests that the overlapping region may contain a minimal CRM (see figure in (1)). These overlaps can arise from RCs that were assayed in different publications and therefore are only discovered through integrated curation in REDfly; this is an increasingly common occurrence as greater numbers of RCs are tested in high-throughput assays. Note that because iCRMs have no direct empirical evidence supporting their function, they are not considered RCs by REDfly.

### Predicted CRMs

While REDfly's emphasis remains on empirically-validated regulatory sequences, we recognize that both genomic and computational methods have in recent years led to a great many regulatory element predictions, for instance based on ChIP-seq, ATAC-seq, or machine-learning (50). For researchers interested in the regulation of a specific gene, there is obvious value in being able to quickly assess whether any CRMs have been predicted in its locus. Therefore, we recently introduced a new data class, the *predicted CRM* (pCRM). Like iCRMs, pCRM records appear in a separate tab of the ‘results’ window. At present, search, display, and download capabilities for pCRMs are limited, but increased functionality is under development. pCRMs are not explicitly linked to potential target genes, but will be recovered in searches for nearby genes when locus-based searching (enabled by default; see below) is employed.

### Additional TFBS classes

We have continued to expand the annotation of TFBSs by curating those confirmed using yeast one-hybrid assays, MARE/MITOMI (51) and surface plasmon resonance (SPR), along with our previously-curated classes of EMSA and DNaseI footprinting.

### Discontinuation of information on syntenic relationships

REDfly v3.0 introduced information on the conservation of local synteny between CRMs and the transcription start sites (TSS) of their respective target genes. Although conservation of synteny provides support for the annotated CRM-target gene relationship, the effort of assessing this for each new CRM has become disproportionate with the utility of this feature and the needs of our user base. Therefore, as of REDfly v5.4.2 we have removed this feature from the ‘basic information’ tab of the detailed record view and are no longer determining whether newly-added CRMs maintain synteny with their target genes. Legacy information on syntenic state for older records can be obtained by request.

## NEW RECORDS

The most significant change in REDfly since our last published description is the growth in curated data (Table 1). Thanks in large part to the increased feasibility of medium- and high-throughput CRM discovery screens (50), as of September 2018 the number of annotated RCs in REDfly had grown over 17-fold, with a 2.4-fold increase in the number genes with at least one associated CRM. A more modest 1.3× increase occurred for annotated TFBSs, while the number of curated publications almost doubled. We expect these numbers to continue to increase dramatically over the next year, as we fold in the approximately 7000 RCs from the Janelia Farms collection (52) and continue to work through our backlog of ∼300 papers. All regulatory features are stored in REDfly as nucleotide sequences and automatically mapped to both release 6 (dm6/August 2014) and release 5 (dm3/April 2006) genome coordinates, using BLAT (53).

One consequence of the growth in large CRM-discovery screens is that regulatory sequences are not always easily matched to their target genes. In cases where authors have not assigned a target gene and where the assignment is not obvious, target genes are listed as ‘unspecified.’ Although initially a minor category, RCs with unspecified target genes now comprise over 35% of the total. Thus, while fewer than 6% of *Drosophila* genes have an explicitly associated CRM, the number of genes for which an associated CRM resides in the database is probably substantially higher. Nevertheless, these numbers suggest that extensive discovery, characterization, and curation of CRMs still remain to be performed. To ensure that RCs annotated with ‘unspecified’ targets are not missed when searching the database, we have implemented ‘locus-based’ searching (see below) as the default option.

## USER INTERFACE

In November 2017, we overhauled the REDfly site to provide a more contemporary look and feel. The web interface is now fully adaptive and should provide a similar user experience on a desktop, tablet, or mobile device. Display of search results remains essentially unchanged, with a results summary pane located directly below the search pane and multiply-tabbed detailed results available by clicking on a row of the results summary table. However, several new search options have been implemented as default settings.

### Locus-based searching

The simplest way to search REDfly, and the entry point for the majority of users, is to enter a gene name using the drop-down list in the ‘Gene Name/FBgn’ box. This worked well for early versions of REDfly, where almost every RC record was explicitly associated with a target gene. However, as REDfly has grown and as high-throughput assays have begun testing large numbers of RCs whose target genes are unknown or uncertain, searching by gene name runs the risk of missing regulatory elements lying near to a gene of interest but annotated as having an ‘unspecified’ target, or which are associated with a different nearby gene. The default search behavior has therefore been set to search ‘by locus,’ which will retrieve all records with the current gene name, but also all other RCs and TFBSs within a defined region surrounding the named gene. The size of this retrieval locus defaults to 10 000 bp upstream and downstream of the named gene, but can be customized using the ‘Search Range Interval’ box in the Advanced Search pane. Searching can be toggled from ‘by locus’ to ‘by name’ (which will restrict results to only RCs/TFBSs specifically annotated with the entered gene name) using the buttons below the ‘Gene Name/FBgn’ box.

### ‘Exclude cell culture only’

High-throughput methods such as STARR-seq (54) are capable of generating thousands of tested RCs, but these assays are restricted to a single cell type (e.g. S2 or Kc167 cells). In order to prevent search results from being dominated by RCs with proven function in only a single cell type, REDfly searches by default will not return Reporter Construct/CRM records discovered exclusively through cell-culture based assays. However, such results can easily be included by unchecking the ‘Exclude Cell Line Only’ box to the right of the ‘Search’ button. Users interested in results from a specific cell type can search for that cell type using the ‘Ontology/Expression Term’ search box under Advanced Search.

### A note on ‘Notes’

Various features of curated REDfly data, such as reporter gene expression intensity, gene expression regulated in response to specific environmental conditions, or aspects of expression pattern that are not easily describable using the *Drosophila* anatomy ontology, do not fit easily into the specific data fields available for each record. Some of these will be addressed by an expanded REDfly data model currently under development (see below). Meanwhile, such data can be found in the ‘Notes’ tab available in each detailed record view. The notes provide free-text elaboration on the core data for each record. Importantly, this field also holds supplementary citation data, for instance where a subsequent study adds to what is known about a previously-described CRM, as we transition to a data model where multiple citations can be associated with a single record. While this information may be of less use for bulk studies of large numbers of CRMs, researchers interested in details about a specific RC/CRM or TFBS are especially encouraged to view the Notes field for the most complete information about each regulatory sequence.

## RELEASES AND VERSIONING

As of REDfly v5.0, REDfly moved away from a continuous data release policy to intermittent versioned releases. This provides users with a simpler way of keeping track of changes to the REDfly data. Recently, we have separated the way that updates to REDfly content and updates to the database codebase are versioned. Version numbers now reflect changes to the codebase and database features, whereas content updates are listed by date in the website header. We anticipate data updates at approximately bimonthly intervals, at which time we will also update gene names and IDs, the anatomy ontology, and other data from external sources such as FlyBase (55).

## COMMUNITY INTERACTION

### Dissemination

REDfly has adopted Twitter as our primary means of communicating with the user community (@REDfly_database). Twitter has a strong user base in the scientific community and provides a convenient way for disseminating information on updates, notable new features, and occasional performance issues or server downtime.

### Author feedback

To help insure the accuracy of our regulatory element annotation, we have implemented an automated author feedback system for new data entries. Upon completion of curation of a paper, an email is automatically sent to the corresponding author with a summary of the extracted information as it will appear in REDfly. Authors then have the option of simply checking an ‘all OK’ box and replying to the email, or sending detailed corrections if deemed necessary. As on average there is a lag of approximately one month between curation and data release, this provides the curators ample time to make corrections based on author feedback.

### Sharing with other databases

REDfly data are shared with both FlyBase (55) and FlyMine (56), providing easy community access to *Drosophila* regulatory sequence data. FlyBase imports the REDfly CRM data into the *Drosophila* genome annotation, where it makes up over 85% of the currently-included ‘regulatory_regions’ and ‘enhancers.’

## IMPLEMENTATION CHANGES

The size and complexity of the REDfly application continues to grow as we include more features and computed entities. To help manage the complexity of the codebase, we have organized it into a more flexible and maintainable structure using the Command-Query Responsibility Segregation (CQRS) paradigm (‘Command–query separation,’ *Wikipedia, The Free Encyclopedia*, https://en.wikipedia.org/w/index.php?title=Command%E2%80%93query_separation&oldid=849207740 [accessed 4 September 2018]). While similar to our previous paradigm—Model View Controller (MVC)—CQRS separates read and write operations on the data model allowing for more streamlined application logic at the controller level and improves support for asynchronous operations, such as simultaneous querying of multiple external data sources. This is beneficial for supporting our curation workflow in cases where a curator uploads a large number of new entries at once, as it allows us to efficiently query several disparate data feeds to gather necessary related information.

The database implementation has also been modified to improve query response times for the user to mitigate searching over REDfly's larger and more complex data set, and to more easily support the curation of new data fields while work is underway on the user interface elements necessary to make these data available to the REDfly users. Two separate databases are now used: a curation and search database. The curation database is optimized for the workflow of uploading, verifying, and editing data entries. It also supports the addition of data elements that are being collected but have not yet been released to the end user such as developmental staging information (see below). The search database, on the other hand, is optimized for query speed and structured to support the current search interface without requiring any changes to the search code until features are ready for release. A simple adapter (e.g. set of SQL queries) automatically creates the search database from the curation database by implementing a database projection (‘Projection (relational algebra),’ *Wikipedia, The Free Encyclopedia*, https://en.wikipedia.org/w/index.php?title=Projection_(relational_algebra)&oldid=854572829 [accessed 4 September 2018]), essentially selecting specific information in the proper format required by the search implementation.

## FORTHCOMING DEVELOPMENTS

REDfly is engaged in a major development effort, and a number of important new features are slated to be introduced in the coming year. Chief among these is a long-awaited update to the basic data model for Reporter Constructs. Currently, RCs that are positive for expression are annotated only with a list of expression patterns. Starting with REDfly v6.0, expression data will be annotated with an expanded list of attributes (Figure 1B). Every listed expression pattern will be linked with the stage(s) of development at which this expression is observed, and an indicator for whether that expression pattern is consistent with or ectopic to that of the assigned target gene. Sexually dimorphic expression differences will be captured by assigning each listed pattern to either males, females, or both sexes. Incorporation of terms from the Gene Ontology (57,58) will allow for proper annotation of regulatory elements that respond to specific signals or environmental cues (e.g. wound healing, hypoxia). Moreover, RCs will be able to be associated with more than one ‘evidence type’ to better capture the range of experimental evidence for each regulatory sequence. For RCs where additional functional information has been published subsequent to the initial report of the construct, secondary PMIDs (citation data) will be able to be associated with the record, to better credit the researchers who have contributed to characterization of the regulatory element. All of these attributes will be fully available for sophisticated searching utilizing an improved search interface. iCRMs and pCRMs will also be made fully searchable.

## CONCLUSION

Fitting for a resource entering its Bar Mitzvah year, REDfly has matured from a simple curation of a few hundred CRMs into a comprehensive warehouse for *Drosophila cis*-regulatory data. Both a new data model and new search capabilities are under development that will greatly enhance our capability to capture the fine details of gene regulation and the ability of our users to easily extract the most useful information from our records. As a comprehensive, unbiased database of experimentally-verified regulatory elements, REDfly will continue to be available as a resource to aid in studies of *Drosophila* and other insect gene regulation; to help develop and validate methods that can be applied to invertebrate and vertebrate systems alike; and for researchers in the genomics, transcription, developmental biology, and evolutionary biology communities. REDfly is freely accessible without restrictions at http://redfly.ccr.buffalo.edu.
